# Dextromethorphan attenuated the higher vulnerability to inflammatory thermal hyperalgesia caused by prenatal morphine exposure in rat offspring

**DOI:** 10.1186/1423-0127-18-64

**Published:** 2011-08-23

**Authors:** Pao-Luh Tao, Chien-Fang Chen, Eagle Yi-Kung Huang

**Affiliations:** 1 Division of Mental Health and Addiction Medicine, Institute of Population Health Sciences, National Health Research Institutes, 35 Keyan Road, Zhunan, Miaoli County 350, Taiwan; 2 Department of Pharmacology, National Defense Medical Center, Taipei, Nei-Hu 114, Taiwan

## Abstract

**Background:**

Co-administration of dextromethorphan (DM) with morphine during pregnancy and throughout lactation has been found to reduce morphine physical dependence and tolerance in rat offspring. No evidence was presented, however, for the effect of DM co-administered with morphine during pregnancy on inflammatory hyperalgesia in morphine-exposed offspring. Therefore, we attempt to investigate the possible effect of prenatal morphine exposure on the vulnerability to hyperalgesia and the possible therapeutic effect of DM in the present study.

**Methods:**

Fifty μl of carrageenan (20 mg/ml) was injected subcutaneously into the plantar surface of the right hind paw in p18 rats to induce hyperalgesia. Mean paw withdrawal latency was measured in the plantar test to index the severity of hyperalgesia. Using Western blotting and RT-PCR, the quantitative analyses of NMDA receptor NR1 and NR2B subunits were performed in spinal cords from different groups of animals.

**Results:**

In the carrageenan-induced hyperalgesia model, rat offspring passively exposed to morphine developed a severe hyperalgesia on postnatal day 18 (p18), which also had a more rapid time course than those in the controls. Co-administration of DM with morphine in the dams prevented this adverse effect of morphine in the offspring rats. Western blot and RT-PCR analysis showed that the levels of protein and mRNA of NMDA receptor NR1 and NR2B subunits were significantly higher in the lumbar spinal cords of rats (p14) exposed to prenatal morphine; the co-administration of DM could reverse the effect of morphine on NR1 and attenuate the effect on NR2B.

**Conclusions:**

Thus, DM may have a great potential in the prevention of higher vulnerability to inflammatory thermal hyperalgesia in the offspring of morphine-addicted mothers.

## Background

Growth retardation, delayed motor development and behavior abnormalities have been proposed in offspring of heroin-addicted mothers [1]. Infants passively exposed to morphine through their addicted mothers easily developed morphine withdrawal syndrome after birth, and even needed intensive care [2-4]. In our previous studies, we observed that many adverse effects caused by prenatal exposure of morphine could be prevented by the co-administration of dextromethorphan (DM) in morphine-dependent rat dams [5,6]. However, the possible impacts of prenatal exposure of morphine on the vulnerability to hyperalgesia have never been examined. In humans, the liability to inflammatory hyperalgesia is often affected by acquired physical conditions and social factors in offspring from morphine-addicted mother [7]. Thus, we attempted to investigate the possible effects of prenatal exposure to morphine on the vulnerability to hyperalgesia in a rat model. In addition, the possible protective effect of the co-administered DM was also tested.

Being a non-competitive antagonist at the glutamatergic NMDA receptors, DM is thought to exert many of its pharmacological actions through the blockade of NMDA receptor [8], although DM was reported to act at the other targets (e.g. nicotinic α3β4 receptors and sigma receptors) as well [9,10]. Activation of the NMDA receptors has been implicated in the development of inflammatory hyperalgesia and the regulation of rewarding-related mesolimbic pathway in many reports [11-13]. Therefore, we speculated that the prenatal administration of morphine and DM to the dams may affect the development of the neural systems which will be functionally correlated with hyperalgesia in the offspring. In order to investigate the vulnerability to hyperalgesia, we used a plantar test in rats with intraplantar carrageenan-induced inflammatory hyperalgesia to perform quantitative verification. These behavioral experiments were carried out on the rats which were the offspring from the morphine-dependent dams.

In the present study, we first demonstrated that the prenatal exposure of morphine could increase the vulnerability to inflammatory hyperalgesia in the offspring. Our biochemical results showed a clear increase of the NR1 and NR2B subunits of NMDA receptors in the spinal cord from the offspring (18 days old; p18) of morphine-treated mother. This could provide some neural developmental evidence which may be related to the higher vulnerability to inflammatory hyperalgesia. This adverse effect of prenatal morphine exposure could be prevented by the co-administration of DM in the dams. Overall, our results highlighted the possible adverse effect of prenatal morphine exposure that is seldom noticed. DM may also have a great therapeutic potential in the prevention of the adverse effect.

## Materials and methods

### Animals

Adult female Sprague-Dawley rats were purchased from the National Experimental Animal Centre, Taipei, Taiwan. The animals were housed one or two in a cage, in a room maintained at a temperature of 23 ±2°C with a 12 h light-dark cycle. Food and water were available *ad libitum *throughout the experiment. Rats were randomly separated into four groups. Rats received subcutaneous (s.c.) injection of saline (Control group), morphine (M group), morphine + dextromethorphan (M + DM group) and dextromethorphan (DM group) twice per day (9 AM and 5 PM) and progressively increased with 1 mg/kg at 7-day intervals from a beginning dose of 2 mg/kg for both morphine and dextromethorphan. The rats were mated on day 8 and the drug administration was continued during pregnancy. After rat offspring were born, the dams were housed separately in individual cages and the injections of drugs into the dams were stopped. Four groups of neonatal rats of either sex aged 18 days (p18) were used for the plantar test. P14 rats were used for Western blot and RT-PCR analysis. The care of animals was carried out in accordance with institutional and international standards (Principles of Laboratory Animal Care, National Institutes of Health), and the protocol was approved by the Institutional Animal Care and Use Committee of National Defense Medical Center, Taiwan, R.O.C.

### Determination of carrageenan-induced thermal hyperalgesia

P18 rats from four groups were used for plantar tests. Fifty μl of carrageenan (20 mg/ml) was injected subcutaneously via a 28-G needle into the plantar surface of the right hind paw. A plantar analgesiometer (7370, Ugo Basile, Italy) was used to index thermal hyperalgesia [14]. Mean paw withdrawal latency in response to the stimulus from a focused beam of I.R. light served as the measure of thermal nociception. Cut-off time of the paw withdrawal latency was set at 10 sec to prevent thermal injury. Animals were all injected with carrageenan on the right hind paw that was subjected to the determination of paw withdrawal latency. The paw withdrawal latencies were measured before carrageenan injection (0 hour), and at the time points after carrageenan injection: 3, 6, 9, 12, 24, 48 hours. The paw withdrawal latency of each rat was tested for three times at each time point. Two closer values of latencies were selected and averaged as the final data to be used. The paw withdrawal latencies were calculated and converted to the percentage of the basal latency for comparison.

### Western blot analysis

P14 rats from four groups were sacrificed by decapitation, and their lumbar (L1 to L6) spinal cords were quickly dissected. These tissues were immediately frozen in liquid nitrogen and kept at -80°C until use. The tissues were disrupted by homogenization on ice in lysis buffer [Tris-HCl 0.05 M, EDTA 5 mM, NaCl 0.15 M, Triton X-100 1%, aprotinin 0.5 μg/ml, leupeptin 0.5 μg/ml, phenylmethanesulfonyl fluoride (PMSF) 30 μg/ml, 1,4-dithiothreitol (DTT) 5 mM]. Tissue lysates were obtained by first centrifugation at 1,000 × g for 10 min, followed by the second centrifugation of the previous supernatant at 35,000 × g for 30 min at 4°C. Protein concentrations were estimated by the BCA protein assay (Pierce, U.S.A.) using bovine serum albumin (BSA) as standards. For Western blot analysis, 25 μg protein of the lysates was dissolved in sample buffer (Tris 0.2 M, SDS 0.8%, glycerol 5%, DTT 3.1 mg/ml, bromophenol blue 0.04 mg/ml), boiled for 5 min, and subjected to SDS-PAGE (10% polyacrylamide). The proteins on the gel were transferred to a polyvinylidene fluoride (PVDF) membrane (FluoroTrans W membrane, pore size: 0.2 μm, PALL Life Sciences, U.S.A.) using a GENIE electrophoretic transfer apparatus (Idea Scientific, U.S.A.). The membranes were incubated with first antibody [1:1K; anti-NMDA NR1 monoclonal antibody, anti-NMDA NR2B polyclonal antibody (Novus Biologicals, U.S.A.), and anti-β-actin monoclonal antibody (Sigma, U.S.A.)] at 4°C over night, and then incubated with secondary antibody [1:2K; anti-rabbit IgG HRP conjugated antibody and anti-mouse IgG HRP conjugated antibody (Alpha Diagnostic, U.S.A.)] for one hour. The bands of proteins were revealed by ECL Western blotting detection kit (Amersham Biosciences, U.K.) and visualized on an X-ray film. Using Kodak Digital Science 1D image analysis software, the optical density of each band was analyzed. The value was calculated as the ratio of the density of corresponding β-actin bands. These ratios were then normalized with the mean ratio obtained from the control group, which was set as 100%.

### Reverse transcription polymerase chain reaction (RT-PCR)

Tissue samples were obtained from the lumbar spinal cords of the p14 rats from four groups. These P14 rats were sacrificed by decapitation, and their lumbar (L1 to L6) spinal cords were quickly dissected. These tissues were immediately frozen in liquid nitrogen and kept at -80°C. Total RNA of the samples was extracted with EZ-10 Spin Column Total RNA MiniPreps Super Kit (Bio Basic Inc., Canada). One μg of total RNA was used per PCR condition. Using the One-Step RT PCR kit (GeneMark, Taiwan, R.O.C.), forty PCR cycles were performed and the products were subjected to agarose gel electrophoresis. Primers used were (5'-3', sense/antisense): TGGGACACGGCTCTGGAAG/TAGGCGGGTGGCTAACTA for NR1, AGCCAAGAGGAGGAAACAGC/ACCTCCACTGACCGAATCTC for NR2B). Using Kodak Digital Science 1D image analysis software, quantitative analysis was performed after scanning of the ethidium bromide-stained agarose gel pictures. The method of quantification was similar to that used in Western blotting, but the bands of GAPDH were used as the internal controls.

### Statistical analysis

The data were all expressed as means ± SEM. One-way ANOVA followed by Newman-Keuls test was employed to examine the statistical significance of the difference between groups.

## Results

### Chronic morphine administration of the dams caused a higher sensitivity to noxious stimuli and more severe inflammatory hyperalgesia in the offspring rats (p18), which could be prevented by the co-administration of DM in the dams

**Figure 1:**
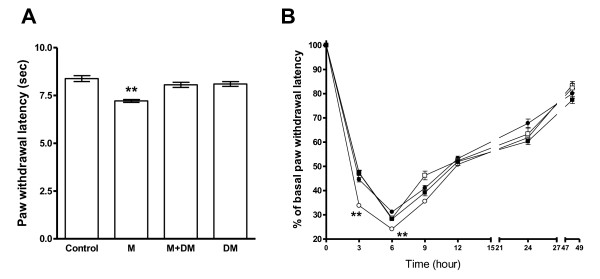
**(*A*) The paw withdrawal latency was obtained by plantar tests before carrageenan injection in the offspring rats (P18) of different groups**. (*B*) The time-course of the hyperalgesia effect induced by carrageenan injection (50 μl, 20 mg/ml). In (*B*), the different marks and lines represent the results from different groups (filled circle: Control, open circle: M, open square: M + DM, filled square: DM). Data are presented as mean ± SEM. One-way ANOVA followed by the Newman-Keuls test was used to analyze the data. (*A*: **p < 0.001 for the morphine group *vs *the control group; *B*: **p < 0.001, for the morphine group *vs *the control group) (Control group: n = 8; M group: n = 8; M + DM group: n = 7; DM group: n = 10).

Before carrageenan injection, p18 rats of the morphine group showed a significantly shorter paw withdrawal latency, when compared with that of the control group (7.2 ± 0.2 sec *versus *8.4 ± 0.4 sec, n = 8, *p *< 0.001) (Figure 1A). P18 rats of the M + DM group showed a similar paw withdrawal latency (8.1 ± 0.4 sec, n = 7) to that of the control group. There was no significant difference of the paw withdrawal latency between the DM group (8.1 ± 0.4 sec, n = 10) and the control group. These data suggest that chronic morphine administration of the dams caused a higher sensitivity to noxious stimuli in the offspring rats. This effect of morphine could be prevented by the co-administration of DM, whereas DM alone did not cause any significant change in the sensitivity to noxious stimuli.

To examine the effect on inflammatory hyperalgesia, paw withdrawal latency was determined on hour 3, 6, 9, 12, 24, 48 after carrageenan injection (Figure 1B). In the control group, the paw withdrawal latency was significantly decreased to 44.5 ± 2.7% and 31.2 ± 2.8% of the basal value (value on hour 0) on hour 3 and hour 6, respectively. This indicates a clear thermal hyperalgesia induced by intra-plantar carrageenan injection in our model. In the morphine group, the paw withdrawal latency was significantly decreased to 33.8 ± 2.4% and 24.1 ± 1.4% of the basal value on hour 3 and hour 6, respectively. These latencies decreased to a lower level in comparison with those of the control group (*p *< 0.001) (Figure 1B). There was no significant difference between the M + DM group and the control group. These results indicate that the offspring rats from the morphine group showed a more severe inflammatory hyperalgesia, which developed more rapidly compared to controls. However, co-administration of DM with morphine in the dams effectively prevented this adverse effect in the offspring rats. But DM alone did not induce any effect on hyperalgesia.

### The quantitative change of NR1/NR2B mRNA and protein expression in the offspring rats from morphine/DM/(morphine + DM)-treated dams

**Figure 2:**
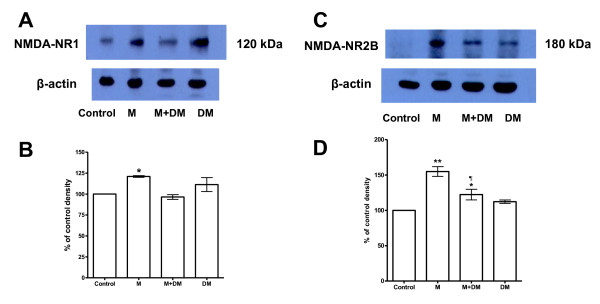
**(*A*), (*C*): The blots show the examples of immunobands against NR1 (*A*) and NR2B (*C*) and β-actin antibodies on the membrane, which was performed on the membrane protein prepared from the lumbar spinal cords of the offspring (P14) rats in different groups**. (*B*), (*D*): The quantitative change of NR1 (*B*) and NR2B (*D*) protein expression. The values of NR1 and NR2B subunits in the control group were arbitrarily set at 100%. Data are presented as mean ± SEM. One-way ANOVA followed by the Newman-Keuls test was used to analyze the data. (*B*: *p < 0.05 for the morphine group *vs *the control group; *D*: **p < 0.001 for the morphine group *vs *the control group; *p < 0.05 for the M + DM group *vs *the control group; ¶*p *< 0.01 for the M + DM group *vs *the morphine group, n = 3).

In the experiments of Western blots, we found that there was an increase of the expression of NR1 and NR2B subunits of the NMDA receptor within the spinal cord from p14 rats of the morphine group (Figure 2A; 2C). When the quantities of the expression of NR1 and NR2B subunits in the control group were set as 100%, the respective values from the morphine group were 121.1 ± 1% in NR1 subunit (*p *< 0.05) and 155 ± 6.9% in NR2B subunit (*p *< 0.001) (Figure 2B; 2D). In the M + DM group, there was no significant difference from that of the control group in NR1 subunit (96.5 ± 27%), but a significant increase of the NR2B subunit (122.3 ± 7.5%, *p *< 0.05) was still observed. Compared with the morphine group, there was a significant lower level of NR2B subunit in the M + DM group (*p *< 0.05) (Figure 2D).

**Figure 3:**
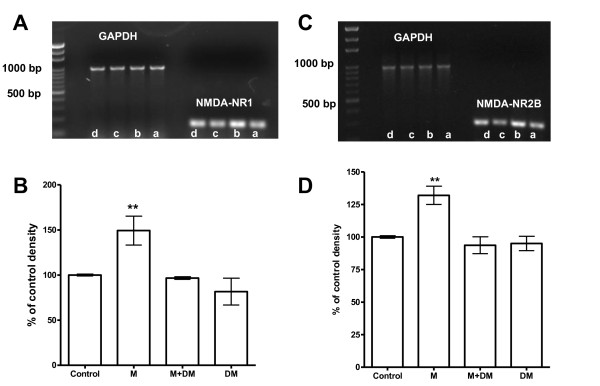
**(*A*), (*C*): An example of RT-PCR results for the levels of mRNA of NMDA receptor NR1 (*A*) and NR2B (*C*) subunits and GAPDH**. (*B*), (*D*): The quantitative RT-PCR results for the levels of mRNA of NMDA receptor NR1 (*B*) and NR2B (*D*) subunit of the offspring rats (P14) in different groups. In (*A*), each white letter at the bottom of the lane represents the result from certain group (a: Control, b: M, c: M + DM, d: DM). The values of NR1 and NR2B subunits in the control group were arbitrarily set at 100%. Data are presented as mean ± SEM. One-way ANOVA followed by the Newman-Keuls test was used to analyze the data. (*B*: **p<0.01 for the morphine group *vs *the control group; *D*: **p < 0.01, for the morphine group *vs *the control group, n = 3).

Consistent with the results of Western blots, the levels of mRNA of NR1 and NR2B subunits were also increased in the spinal cords from p14 rats of the morphine group (Figure 3A; 3C). When the mRNA values of NR1 and NR2B subunits in the control group were set as 100%, the respective values from the morphine group were significantly increased to 149.3 ± 16% in NR1 subunit (*p *< 0.01) and to 132 ± 7% in NR2B subunit (*p *< 0.01) (Figure 3B; 3D). In the M + DM group, there was no significant difference from that of the control group in both NR1 (96.6 ± 1.4%) and NR2B subunits (93.6 ± 6.4%) (Figure 3B; 3D).

## Discussion

Previously, we found that the co-administration of dextromethorphan with morphine to dam rats throughout pregnancy significantly decreased morphine physical dependence and tolerance in their offspring [5]. In the present study, our results suggested that co-administration of DM with morphine during pregnancy could possibly attenuate the vulnerability to inflammatory hyperalgesia in offspring rats from the dam with chronic morphine exposure. In our experiments, we first observed a lower thermal pain threshold in the offspring rats from morphine-addicted mother. But the pain threshold was found to be as same as that of the control group, when DM was co-administered with morphine in the dams. This implied a higher sensitivity to pain was induced by prenatal exposure to morphine in the dams, which could be diminished by the co-administration of DM. Hovious and Peters first demonstrated that chronic maternal exposure to morphine has a significant effect on the effectiveness of analgesic drugs in the offspring rats [15]. Their results showed decreased response latencies in offspring (p25 and p120) from morphine-treated mother in both tail-flick and hot-plate tests. Although it was more significant in female offspring rats, this report suggested that chronic maternal exposure of morphine could possibly increase the sensitivity to pain in the offspring rats. The following studies also confirmed this finding of morphine's prenatal effects [16,17]. Although the dosage and schedule of the maternal morphine administration are different, our current results are consistent with these reports. Moreover, we found that maternal co-administration of DM with morphine could prevent the increase of pain sensitivity in the offspring.

In the present study, we further examined the possible effect of morphine's prenatal effect on inflammatory hyperalgesia. Using carrageenan-induced plantar inflammation and plantar test, we observed a more severe hyperalgesia in offspring from morphine-treated dams. Again, this effect could be prevented by the maternal co-administration of DM with morphine. Since maternal treatment of DM itself did not cause any effect on the antinociceptive response and hyperalgesia, DM may be of great therapeutic potential in correlation with the lessening of adverse effects in offspring from morphine-addicted female patients. In view of the age of offspring rats to be tested, we used p18 rats of either sex in the plantar tests. This is also the age of rats showing most significant difference in nociceptive sensitivity between control and prenatal morphine-treated group, which was reported by Zhang and Sweitzer [17]. In this recent report, they found that there was no difference of nociceptive sensitivity between groups at the age over p50.

In search of the possible underlying mechanisms, we examined the level of protein and mRNA of NMDA receptor NR1 and NR2B subunits in the lumbar spinal cords of offspring rats (p14) from different groups. The data showed that the level of NMDA receptor NR1 and NR2B subunits were significantly higher in the morphine group, whereas the maternal co-administration of DM could reverse the effect on NR1 but attenuate the effect on NR2B. Moreover, the prenatal exposure of DM alone did not change the expression of NR1 and NR2B. Interestingly, the mRNA data for NR2B seem to show that the maternal co-administration of DM could totally reverse the effect caused by morphine, which is different from the decrease of NR2B at the protein level. This may be due to the low sensitivity of RT-PCR quantification or the difference between the level of mRNA and protein. The correlation between the NMDA-receptor system and hyperalgesia has been demonstrated since both systemic and intrathecal injections of morphine, specific (MK-801) and nonspecific NMDA-receptor antagonists (DM) could cause a significant reduction of hyperalgesia [18-23]. Moreover, injury-induced hyperalgesia, morphine tolerance, and changes in NR1 mRNA produced by chronic morphine were found to be prevented by the blockade of NMDA receptors in the spinal cord dorsal horn [24-26]. Therefore, the spinal NMDA-receptors were regarded as a functionally important pronociceptive system which was also correlated with hyperalgesia [27]. Although the detailed mechanism of maternal DM to suppress NMDA-receptor expression was unknown, our results provide some possible biochemical evidence in connection with our behavioral findings. Nevertheless, the increase of the expression of NMDA receptor NR1 and NR2B subunits should not be regarded as the sole reason for the higher vulnerability to inflammatory thermal hyperalgesia in prenatal morphine-exposed offspring. Many other biochemical and physical changes could be also involved in the generation of this higher vulnerability. For example, the endogenous opioid peptides and opioid receptors could be changed for their quantities or sensitivities by prenatal morphine exposure. Certainly, the opioid system may contribute to the higher vulnerability to hyperalgesia. This requires further investigations on the possible change of the opioid system. So far, we were only able to conclude that the quantitative change of the NMDA receptor subunits may play a role.

Regarding to the pharmacological target of DM to reduce the adverse effects of prenatal morphine, many behavioral studies revealed that the NMDA receptor antagonism of DM is important for its action to potentiate the antinociceptive effect of morphine in rats [28,29]. Although a recent clinical report indicated that MorphiDex (morphine sulfate/dextromethorphan hydrobromide combination) failed to enhance opioid analgesia or reduce tolerance [30], the possible contribution of NMDA receptor blockade by DM could be still of importance in its action to regulate pain. Depending on the dose and the species, NMDA receptor antagonists showed various effects to attenuate pain/nociception in different animal models [31,32]. Therefore, DM may possibly act through the blockade of NMDA receptors to affect morphine-induced higher vulnerability to hyperalgesia in offspring from morphine-treated dams. However, the relevant mechanisms of prenatal DM remain to be tested.

## Conclusions

In summary, the present study provides behavioral and biochemical evidences in neonatal rats passively exposed to morphine throughout embryo stages, which suggest that they could be more susceptible to developing many adverse effects, such as inflammatory hyperalgesia. Therapeutically, DM could reverse this adverse effect caused by prenatal morphine. The current results also implied the possible biological change in the CNS of offspring from morphine-addicted mother in humans. Moreover, the therapeutic potential of DM was further highlighted; especially our recent report also indicated the ability of DM to reduce morphine-induced hyperprolactinemia in female rats at different reproductive stages [33].

## Competing interests

The authors declare that they have no competing interests.

## Authors' contributions

CFC carried out the experiments. PLT and EYH conceived of the study, and participated in its design and coordination. All authors read and approved the final manuscript.
